# Homozygous Deletion of Six Olfactory Receptor Genes in a Subset of Individuals with Beta-Thalassemia

**DOI:** 10.1371/journal.pone.0017327

**Published:** 2011-02-24

**Authors:** Jessica Van Ziffle, Wendy Yang, Farid F. Chehab

**Affiliations:** Department of Laboratory Medicine, University of California San Francisco, San Francisco, California, United States of America; Center for Genomic Regulation, Spain

## Abstract

Progress in the functional studies of human olfactory receptors has been largely hampered by the lack of a reliable experimental model system. Although transgenic approaches in mice could characterize the function of individual olfactory receptors, the presence of over 300 functional genes in the human genome becomes a daunting task. Thus, the characterization of individuals with a genetic susceptibility to altered olfaction coupled with the absence of particular olfactory receptor genes will allow phenotype/genotype correlations and vindicate the function of specific olfactory receptors with their cognate ligands. We characterized a 118 kb β-globin deletion and found that its 3′ end breakpoint extends to the neighboring olfactory receptor region downstream of the β-globin gene cluster. This deletion encompasses six contiguous olfactory receptor genes (OR51V1, OR52Z1, OR51A1P, OR52A1, OR52A5, and OR52A4) all of which are expressed in the brain. Topology analysis of the encoded proteins from these olfactory receptor genes revealed that OR52Z1, OR52A1, OR52A5, and OR52A4 are predicted to be functional receptors as they display integral characteristics of G-proteins coupled receptors. Individuals homozygous for the 118 kb β-globin deletion are afflicted with β-thalassemia due to a homozygous deletion of the β-globin gene and have no alleles for the above mentioned olfactory receptors genes. This is the first example of a homozygous deletion of olfactory receptor genes in human. Although altered olfaction remains to be ascertained in these individuals, such a study can be carried out in β-thalassemia patients from Malaysia, Indonesia and the Philippines where this mutation is common. Furthermore, OR52A1 contains a γ-globin enhancer, which was previously shown to confer continuous expression of the fetal γ-globin genes. Thus, the hypothesis that β-thalassemia individuals, who are homozygous for the 118 kb deletion, may also have an exacerbation of their anemia due to the deletion of two copies of the γ-globin enhancer element is worthy of consideration.

## Introduction

The β-globin gene locus lies between two olfactory receptor (OR) gene clusters [1] and is subject to multiple deletions that cause β-thalassemia, a transfusion-dependent anemia. However, only three deletions were reported to extend to either olfactory receptor region. Two deletions break in the upstream olfactory receptor region, whereby one removes two [2] and the other four olfactory receptor genes [3]. Because both of these deletions also take out the embryonic epsilon gene, they have not been observed in homozygous individuals due to their presumed embryonic lethality. The third deletion, also occurring in a heterozygous individual and not reported in a homozygous state, extends to the downstream olfactory receptor region where it deletes a single olfactory receptor gene that contains a γ-globin enhancer [4], [5].

A large deletion originally reported in Filipinos and known as the 45 kb deletion [6], [7] has a defined upstream breakpoint between the δ and β-globin genes, however its 3′ end breakpoint had not yet been precisely characterized and remains unknown. In this communication, we report on the accurate characterization of the downstream breakpoint of the 45 kb deletion and uncover that it actually consists of 118 kb and extends to the downstream olfactory receptor region where it encompasses six olfactory receptor genes and the previously characterized γ-globin enhancer. As individuals homozygous for the 118 kb deletion are widespread in Southeast Asia, the possibility arises they could have altered olfaction due to the homozygous deletion of the olfactory receptor genes and an exacerbation of their anemia due to the homozygous deletion of the γ-globin enhancer.

## Results

### Characterization of the deletion breakpoint

**Figure 1:**
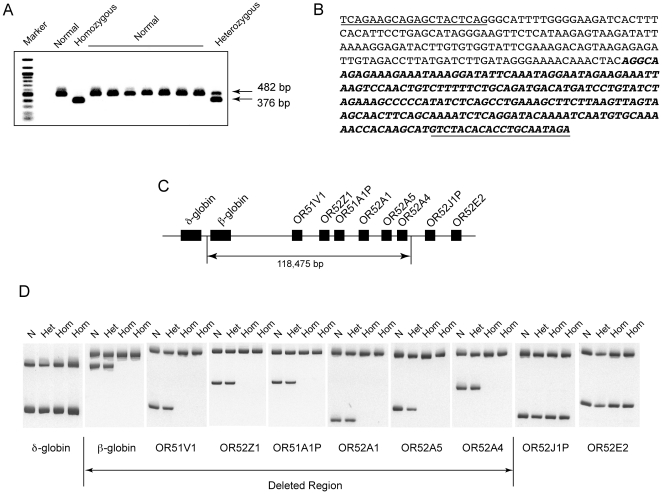
Characterization of the 3′ breakpoint of the 118 kb deletion. (**A**) Amplification of the 376 bp deletion breakpoint in heterozygous and homozygous individuals with the 118 kb deletion. The normal and mutant alleles are denoted by the 482 bp and 376 bp amplicons, respectively. (**B**) DNA sequence of the 376 bp breakpoint sequence. The 5′ end of the breakpoint at the δβ-globin intergenic region is un-bolded while its 3′ end is bolded and is at the olfactory receptor cluster breakpoint. The sequences of PCR primers are underlined at both ends. (**C**) Physical map of the region that separates the two breakpoints shows that it consists of 118,475 bp and encompasses 6 olfactory receptor (OR) genes. (**D**) Absence of PCR amplification from the β-globin gene and OR genes OR51V1, OR52Z1, OR51A1P, OR52A1, OR52A5, OR52A4 in two individuals homozygous for the 118 kb deletion. PCR analysis of normal (N), heterozygous (Het) and homozygous (Hom) individuals shows the absence of specific amplicons in homozygous individuals only. Amplicons from δ-globin, OR52J1P and OR52E2, which are outside the deleted region are present in all individuals. The top band in each gel represents an internal control (IC) amplicon from the cystic fibrosis gene, which is amplified in the same reaction to demonstrate successful amplification, whenever the target amplicon is deleted.

Determination of the DNA sequence from the 376 bp amplicon spanning the deletion breakpoint (Fig. 1A, 1B) and its subsequent alignment to the latest DNA sequence of the human genome (NCBI Bulid 37.1) revealed that 166 bp were homologous to the intergenic region between the δ and β-globin genes, consistent with a previously reported 5′ breakpoint end [7]. However, the remaining 210 bp, which correspond to the 3′ downstream breakpoint, mapped between the OR genes OR52A4 and OR52J1P. The two breakpoints were 118,475 bp apart and the deletion spanned six OR genes: OR51V1, OR52Z1, OR51A1P, OR52A1, OR52A5, OR52A4 (Fig. 1C).

### Genotyping at the OR gene locus of patients homozygous for the 118 kb deletion

To confirm the extent of this deletion that we correctly rename as the 118 kb deletion rather than the 45 kb deletion, we performed PCR genotyping at each of the six suspected deleted OR genes in β-thalassemia individuals homozygous for the 118 kb deletion. As expected, these patients failed to amplify a PCR amplicon from the β-globin gene and from any of the six OR genes (Fig. 1D), thus confirming the homozygous deletion of OR51V1, OR52Z1, OR51A1P, OR52A1, OR52A5, OR52A4.

### Topology and expression of the ORs spanned by the 118 kb deletion

**Figure 2:**
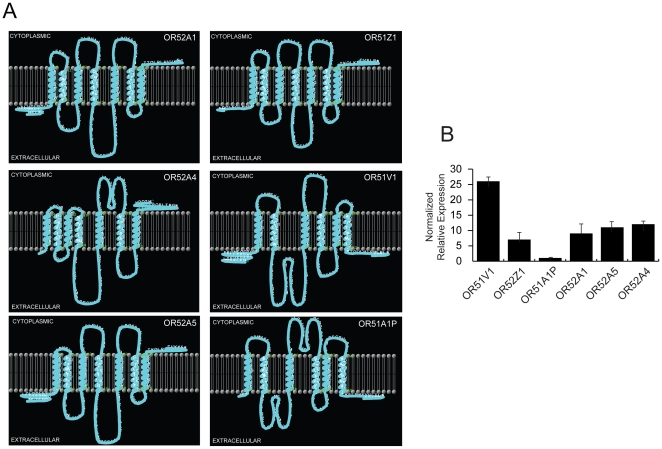
Topology of OR proteins and gene expression. (**A**) Computer prediction models of the 6 OR proteins encompassed by the 118 kb deletion. Characteristic features of functional GPCRs are shown for OR52A1, OR52A5, OR52A4, OR52Z1, including an extracellular amino-terminus, 7 TMHs, and a cytoplasmic carboxy-terminus. OR51V1 and OR51A1P have 6 TMHs and two extracellular termini. (**B**) Relative expression levels of OR52A1, OR52A4, OR52A5, OR52Z1, OR51V1, OR51A1P mRNA in brain by qPCR. All amplifications were run in triplicates for each OR and normalized to an actin internal control mRNA. Displayed data (means and standard deviations) are normalized to OR51A1P.

Algorithm-based topology predictions of the 6 OR proteins encompassed by the 118 kb deletion revealed that OR52Z1, OR52A1, OR52A5, and OR52A4 span each seven transmembrane helices (TMHs) that are flanked by extracellular amino and intracellular carboxy termini. However, OR51V1 and OR51A1P span six TMHs only and are flanked by two extracellular termini (Fig. 2A). Thus, OR52Z1, OR52A1, OR52A5 and OR52A4 are predicted to be functional genes whereas OR51V1 and OR51A1P are pseudogenes. Relative expression levels of the six OR genes in human brain revealed wide expression levels. Specifically, OR51V1 was expressed 25-fold more than OR51A1P, which was expressed at the lowest level of all genes. The four functional genes were expressed 5-fold less than OR51V1 but relatively equally (Fig. 2B).

## Discussion

The olfactory receptor repertoire represents one of the largest human gene families [8] comprising 388 functional genes and 414 pseudogenes [9]. ORs are the largest family of G-protein coupled receptors and their classification into genes and pseudogenes relies on the presence in their encoded protein sequences of an extracellular amino terminus for ligand binding, seven transmembrane helices for membrane potential and a carboxy intracellular terminus for signal transduction. In this context, OR52Z1, OR52A1, OR52A5 and OR52A4 are functional genes and their absence in β-thalassemia individuals homozygous for the 118 kb deletion raises the question as to whether it would result in altered olfaction. This association would be particularly revealing as it will provide an approach to link particular ligands to their cognate receptors. As these studies require the recruitment of reasonably sized cohorts of β-thalassemia patients homozygous for the 118 kb deletion, they would most appropriately be carried out on patients from the Philippines, Malaysia and Indonesia where the combined population of about 300 million individuals carries the highest β-thalassemia incidence worldwide [10]. In fact, the 118 kb deletion occurs at about 3%, 7.8% and 46% in Indonesia, Malaysia and the Philippines, respectively [11], [12], [13] and the overall β-thalassemia carrier rate in these countries ranges from 6–10% in Indonesia [11], [14], 3.5–4.5% in Malaysia [15] and 1–2% in the Philippines [7], [13].

The association of olfactory impairment is well-documented in a congenital disorder such as Kallmann syndrome [16] in which olfactory receptors are lacking and in neurodegenerative disorders such as Alzheimer's disease, mild cognitive impairment [17] and Parkinson's disease [18] that are accompanied with a progressive impairment in the sense of smell. Although none of these is attributed directly to specific OR gene defects, the inability of individuals to smell low levels of isovaleric acid was linked to a nonsense mutation in the olfactory receptor OR11H7P [19]. Heterozygous OR deletions or single gene OR homozygous deletions other than the one reported here, were noted in cell lines [20] but not in individuals. To our knowledge, this is the first example of a homozygous deletion that encompasses multiple olfactory receptor genes and it is coincidental that it occurs in homozygous β-thalassemia individuals afflicted with a transfusion-dependent severe anemia. This finding opens the possibility that other diseases with an underlying causative gene, which is subject to deletions and which is located near an olfactory receptor region could recapitulate a similar situation. For example, mutations in the human aspartoacylase gene, which is flanked by an olfactory receptor rich-region on chromosome 17p13.3, cause Canavan disease. A rare 190 kb deletion spanning the aspartoacylase gene was found in two homozygous individuals and although the deletion extended to the adjoining OR gene region, it did not encompass any OR gene and stopped short of OR1D2 (also called OR17-4) [21].

Whether β-thalassemia individuals homozygous for the 118 kb deletion have altered olfaction needs to be ascertained to determine the impact on the overall management of their disease. Although we have not found any evidence in the recent scientific literature that the olfactory receptors encompassed by the 118 kb deletion have been deorphaned, it was noted that most human homologs of rodent ORs for n-aliphatic odorants are found at a single locus on chromosome 11p15 [22], which is also the location of the β-globin gene [23]. Thus, any olfactory screen for individuals homozygous for the 118 kb deletion ought to take into account the presence of n-aliphatic odorants in the assay. The commercial availability of the “Sniffin' Sticks” assay [24], a widely used clinical test for olfaction, should ease the implementation of such a screening initiative. The issue whether absence of four OR genes among a total repertoire of 388 functional OR genes could cause an olfactory defect may at first seem unlikely but worth exploring. Impairment of olfactory sensitivities were found in even single OR genes. As indicated earlier, olfactory impairment to isovaleric acid was noted in individuals homozygous for a nonsense mutation in the OR gene OR11H7 [19] while individuals homozygous for two amino acid substitutions (R88W and T133M) in OR7D4 had an altered sensitivity to androstenone and androstadienone [25]. Thus, it is conceivable that deletion of multiple OR genes in homozygous individuals may result in altered olfactory abilities to specific odorants. A positive association would have a significant impact on the biology of olfactory receptors as only few ligands have been identified for human ORs [19], [26], [27], [28], [29].

Brain-specific expression of the six OR genes encompassed by the 118 kb deletion was variable whereas that of the four functional OR genes was similar and is characteristic of G-proteins coupled receptor gene regulation [30]. Nonetheless, the differential expression of pseudogenes OR51V1 and OR51A1P may be explained on the basis that expressed pseudogenes are likely to have recently emerged [31] and thus retained their expression levels prior to their divergence into non-functional genes. In this vein, OR51V1 appears to have arisen more recently than OR51A1P.

Of particular interest to β-thalassemia is OR52A1, which was previously shown to contain a γ-globin enhancer element that confers continuous expression of the two fetal γ-globin genes [4], [5] that are overexpressed in β-thalassemia and tend to alleviate the anemia by increasing hemoglobin F production. We hypothesize that the absence of this enhancer from β-thalassemia patients homozygous for the 118 kb deletion may contribute to a significant reduction of their compensatory γ-globin gene expression, leading to lower hemoglobin F levels and an exacerbation of their anemia. In support of this hypothesis is a study of 20 Malaysian children homozygous for this deletion, who were described to have a severe anemia [10]. While recent genome-wide association studies aimed at mapping modifier genes which influence clinical severity of sickle cell anemia and Hb E/β^0^-thalassemia revealed linkages to the OR region upstream of the β-globin gene [32], [33], our present studies are consistent with the hypothesis that the OR52A1 [4], [5] locus in the downstream OR region plays an important role in influencing clinical severity in this type of β-thalassemia.

In conclusion, we report on three main findings. First, the occurrence of a homozygous deletion spanning four functional OR genes and two OR pseudogenes in a subset of patients affected with β-thalassemia. This is the first example of a homozygous deletion of four contiguous and functional OR genes in human individuals. Although it remains to be determined whether the homozygous deletion of these ORs actually causes impaired olfaction, its wide occurrence in Southeast Asia should allow such an evaluation using commercially available standardized smell test assays. Second, the deletion of the γ-globin enhancer by the 118 kb deletion raises the suspicion that its absence may exacerbate this β-thalassemia phenotype. Third, the size of this deletion has been confirmed to be 118 kb, rather than 45 kb, and should be referred to as such in the literature and in molecular diagnostic reports.

## Materials and Methods

### Breakpoint amplification and genotyping of individuals with the Filipino deletion

A PCR amplification reaction targeting the deletion breakpoint in normal, heterozygous and homozygous mutant individuals with the Filipino deletion was modified to detect simultaneously the normal and mutant alleles using three PCR primers 45F: 5′ TCA GAA GCA GAG CTA CTC AG 3′; 45R1- 5′ GTC TAT GCA GGT GTG TAG ACA 3′ and 45R2: 5′ CAT TTA GCT CCC ACA CTC CT 3′. The normal and mutant allele amplicons consisted of 482 bp and 376 bp, respectively. Each PCR was carried out in a 25 µl reaction consisting of a Qiagen multiplex mix with 30, 20 and 10 pmol/µl of primers 45F, 45R1 and 45R2, respectively. Cycling and temperature conditions consisted of an initial denaturation at 95°C for 15 min. followed by successive annealing temperatures of 65°C, 63°C, 61°C, 59°C, 57°C, for the first 4 cycles followed by 30 cycles at 55°C. All subsequent denaturation (94°C) and extension (72°C) steps were for 30 sec each. Reactions were then resolved on a 1% agarose gel and visualized under ultraviolet light with SYBR Safe green.

### DNA sequence alignment of the 3′ end deletion breakpoint

The DNA sequence of the mutant amplicon generated in the deletion breakpoint PCR described above was determined by standard dideoxy sequencing and aligned to the Genbank 177.0 release using the National Center for Biotechnology Information (NCBI) Basic Local Alignment Search Tool (BLAST).

### Mapping of the deletion breakpoints

PCR primers for the δ-globin, β-globin and OR genes, OR52E2, OR52A5, OR52A4, OR52A1, OR52Z1, OR51A1P, OR51V1, OR52J1P and the CFTR internal control are shown in Table 1. PCR products were resolved on 8% native polyacrylamide gels and stained with SYBR Safe green.

**Table 1:** PCR primers used for genotyping individuals at each of the specified locus.

Region	Forward primer (5′ to 3′)	Reverse primer (5′ to 3′)	Amplicon size (bp)
δ-globin	GGTTTCTGAGTCAAGACAC	CAGTATTCTATGCCTCTCAT	1051
β-globin	GCTGTCCAATTTCTATTAAAGG	CAGCATAGCAAAACTTTAACCTC	375
OR52E2	CCATCCCTAAGATGCTTGGA	AAAGCCCTCACAAACACACC	231
OR52A5	CACCATCTTTTCCCAGCAGT	GGATTGCAAAGGCAACAAAT	225
OR52A4	GTTGGTGGGCTTGACTTCAT	GAGGAATGGTGGGACCAGTA	247
OR52A1	AATCTGAGCGCAGTCTCCAT	CACATTTGAAGCAAGCAGGA	157
OR52Z1	CATAGCAGCTGTGGTCAGGA	GCCACAGTGAGGCCATAAAT	167
OR51A1P	GCGCTACACAACCATTCTGA	GTGTCTCCACAGGGCAATTT	185
OR51V1	CTTTCCATCCCCTTCTCCTC	AATAGGACTGGGCAATGCAG	229
OR52J1P	GAGTATTGGCAGGCATTGGT3	GAAGCCACAAAAAGCCCATA	196
CFTR Internal control	GCCCGACAAATAACCAAGTGA	GCTAACACATTGCTTCAGGCT	454

### Expression of OR genes

Total human brain RNA was purchased from Clontech and aliquots converted to cDNA. Real-time quantitative PCR was carried out on a Roche LightCycler 480 using brain cDNA, a SYBR Green I PCR mix (Roche) and the same primers listed above for mapping the deletional breakpoint. Data analysis was performed with the QGene Software [34] and the expression results normalized to OR51A1P.

### Transmembrane helices prediction

Membrane topology of the six ORs was predicted using a web-based algorithm (http://www.cbs.dtu.dk/services/TMHMM/) [35] and a stand alone Java-based application [36], which was used to graphically display the models in Fig. 2A. The following NCBI reference sequences were used: OR52Z1 (predicted from NG_004304.6), OR52A1 (NP_036507.2), OR52A5 (NP_001005160.1), OR52A4 (NP_001005222.1), OR51V1 (NP_001004760.2) OR51A1P (predicted from NG_002199.3).

## References

[1] Bulger M, Bender MA, van Doorninck JH, Wertman B, Farrell CM (2000). Comparative structural and functional analysis of the olfactory receptor genes flanking the human and mouse beta-globin gene clusters.. Proc Natl Acad Sci U S A.

[2] Game L, Bergounioux J, Close JP, Marzouka BE, Thein SL (2003). A novel deletion causing (epsilon gamma delta beta) degrees thalassaemia in a Chilean family.. Br J Haematol.

[3] Brantberg A, Eik-Nes SH, Roberts N, Fisher C, Wood WG (2009). Severe intrauterine anemia: a new form of epsilongammagammadeltabeta thalassemia presenting in utero in a Norwegian family.. Haematologica.

[4] Feingold EA, Forget BG (1989). The breakpoint of a large deletion causing hereditary persistence of fetal hemoglobin occurs within an erythroid DNA domain remote from the beta-globin gene cluster.. Blood.

[5] Feingold EA, Penny LA, Nienhuis AW, Forget BG (1999). An olfactory receptor gene is located in the extended human beta-globin gene cluster and is expressed in erythroid cells.. Genomics.

[6] Eng B, Chui DH, Saunderson J, Olivieri NF, Waye JS (1993). Identification of two novel beta zero-thalassemia mutations in a Filipino family: frameshift codon 67 (-TG) and a beta-globin gene deletion.. Hum Mutat.

[7] Waye JS, Eng B, Hunt JA, Chui DH (1994). Filipino beta-thalassemia due to a large deletion: identification of the deletion endpoints and polymerase chain reaction (PCR)-based diagnosis.. Hum Genet.

[8] Buck L, Axel R (1991). A novel multigene family may encode odorant receptors: a molecular basis for odor recognition.. Cell.

[9] Niimura Y, Nei M (2006). Evolutionary dynamics of olfactory and other chemosensory receptor genes in vertebrates.. J Hum Genet.

[10] Thong MK, Rudzki Z, Hall J, Tan JA, Chan LL (1999). A single, large deletion accounts for all the beta-globin gene mutations in twenty families from Sabah (North Borneo), Malaysia. Mutation in brief no. 240. Online.. Hum Mutat.

[11] Setianingsih II, Williamson R, Marzuk S, Harahap A, Tamam M (1998). Molecular Basis of beta-Thalassemia in Indonesia: Application to Prenatal Diagnosis.. Mol Diagn.

[12] Wee YC, Tan KL, Kuldip K, Tai KS, George E (2008). Alpha-thalassaemia in association with beta-thalassaemia patients in Malaysia: a study on the co-inheritance of both disorders.. Community Genet.

[13] Ko TM, Caviles AP, Hwa HL, Liu CW, Hsu PM (1998). Prevalence and molecular characterization of beta-thalassemia in Filipinos.. Ann Hematol.

[14] Sofro AS (1995). Molecular pathology of beta-thalassemia in Indonesia.. Southeast Asian J Trop Med Public Health.

[15] George E, Li HJ, Fei YJ, Reese AL, Baysal E (1992). Types of thalassemia among patients attending a large university clinic in Kuala Lumpur, Malaysia.. Hemoglobin.

[16] Kallmann F, Schoenfeld W, Barrera S (1944). The genetic aspects of primary eunuchoidism.. Am J Ment Def.

[17] Albers MW, Tabert MH, Devanand DP (2006). Olfactory dysfunction as a predictor of neurodegenerative disease.. Curr Neurol Neurosci Rep.

[18] Ansari KA, Johnson A (1975). Olfactory function in patients with Parkinson's disease.. J Chronic Dis.

[19] Menashe I, Abaffy T, Hasin Y, Goshen S, Yahalom V (2007). Genetic elucidation of human hyperosmia to isovaleric acid.. PLoS Biol.

[20] Hasin Y, Olender T, Khen M, Gonzaga-Jauregui C, Kim PM (2008). High-resolution copy-number variation map reflects human olfactory receptor diversity and evolution.. PLoS Genet.

[21] Tahmaz FE, Sam S, Hoganson GE, Quan F (2001). A partial deletion of the aspartoacylase gene is the cause of Canavan disease in a family from Mexico.. J Med Genet.

[22] Malnic B, Godfrey PA, Buck LB (2004). The human olfactory receptor gene family.. Proc Natl Acad Sci U S A.

[23] Morton CC, Kirsch IR, Taub R, Orkin SH, Brown JA (1984). Localization of the beta-globin gene by chromosomal in situ hybridization.. Am J Hum Genet.

[24] Hummel T, Sekinger B, Wolf SR, Pauli E, Kobal G (1997). ‘Sniffin' sticks’: olfactory performance assessed by the combined testing of odor identification, odor discrimination and olfactory threshold.. Chem Senses.

[25] Keller A, Zhuang H, Chi Q, Vosshall LB, Matsunami H (2007). Genetic variation in a human odorant receptor alters odour perception.. Nature.

[26] Schmiedeberg K, Shirokova E, Weber HP, Schilling B, Meyerhof W (2007). Structural determinants of odorant recognition by the human olfactory receptors OR1A1 and OR1A2.. J Struct Biol.

[27] Spehr M, Gisselmann G, Poplawski A, Riffell JA, Wetzel CH (2003). Identification of a testicular odorant receptor mediating human sperm chemotaxis.. Science.

[28] Wetzel CH, Oles M, Wellerdieck C, Kuczkowiak M, Gisselmann G (1999). Specificity and sensitivity of a human olfactory receptor functionally expressed in human embryonic kidney 293 cells and Xenopus Laevis oocytes.. J Neurosci.

[29] Hatt H, Gisselmann G, Wetzel CH (1999). Cloning, functional expression and characterization of a human olfactory receptor.. Cell Mol Biol (Noisy-le-grand).

[30] Lattin JE, Schroder K, Su AI, Walker JR, Zhang J (2008). Expression analysis of G Protein-Coupled Receptors in mouse macrophages.. Immunome Res.

[31] Zhang X, De la Cruz O, Pinto JM, Nicolae D, Firestein S (2007). Characterizing the expression of the human olfactory receptor gene family using a novel DNA microarray.. Genome Biol.

[32] Solovieff N, Milton JN, Hartley SW, Sherva R, Sebastiani P (2010). Fetal hemoglobin in sickle cell anemia: genome-wide association studies suggest a regulatory region in the 5′ olfactory receptor gene cluster.. Blood.

[33] Sherva R, Sripichai O, Abel K, Ma Q, Whitacre J (2010). Genetic modifiers of Hb E/beta0 thalassemia identified by a two-stage genome-wide association study.. BMC Med Genet.

[34] Muller PY, Janovjak H, Miserez AR, Dobbie Z (2002). Processing of gene expression data generated by quantitative real-time RT-PCR.. Biotechniques.

[35] Moller S, Croning MD, Apweiler R (2001). Evaluation of methods for the prediction of membrane spanning regions.. Bioinformatics.

[36] Spyropoulos IC, Liakopoulos TD, Bagos PG, Hamodrakas SJ (2004). TMRPres2D: high quality visual representation of transmembrane protein models.. Bioinformatics.

